# A multidisciplinary assessment of endocrine and morphometric correlates of lactation physiology in southern right whales

**DOI:** 10.1093/conphys/coag014

**Published:** 2026-03-27

**Authors:** Loraine Shuttleworth, Andre Ganswindt, Els Vermeulen

**Affiliations:** Mammal Research Institute, Whale Unit, Department of Zoology and Entomology, Faculty of Natural and Agricultural Sciences, University of Pretoria, Cnr Lynnwood Road and Roper Street, Hatfield, Pretoria 0002, South Africa; Mammal Research Institute, Whale Unit, Department of Zoology and Entomology, Faculty of Natural and Agricultural Sciences, University of Pretoria, Cnr Lynnwood Road and Roper Street, Hatfield, Pretoria 0002, South Africa; Mammal Research Institute, Whale Unit, Department of Zoology and Entomology, Faculty of Natural and Agricultural Sciences, University of Pretoria, Cnr Lynnwood Road and Roper Street, Hatfield, Pretoria 0002, South Africa

**Keywords:** Body condition, capital breeder, cortisol, endocrinology, energetics, glucocorticoids, photogrammetry, thyroid hormone, triiodothyronine

## Abstract

Reproduction in long-lived, iteroparous mammals requires careful allocation of energetic resources. This is especially true in capital breeding species such as the southern right whale (*Eubalaena australis*), where reproductive success depends on body condition, as accumulated reserves must fuel the high energetic demands, especially during lactation. Understanding the physiological mechanisms underpinning maternal energy balance is therefore critical for assessing reproductive capacity and maternal investment strategies. This study combined body condition metrics derived from aerial photogrammetry with endocrine correlates (glucocorticoids and triiodothyronine; hormones associated with energy mobilisation and regulation of metabolism) measured in blubber biopsies to establish baseline measures of metabolic function in lactating southern right whales over the calving season (July–September). Analyses revealed a positive correlation between glucocorticoids and triiodothyronine, strongest in lactating females. A clear decline in both hormones was observed toward the end of the calving season, consistent with reduced maternal metabolic activity and corresponding to slowed calf growth rates. No significant differences in hormone concentrations were detected between demographic groups (*n* = 15 lactating females, *n* = 8 unaccompanied adults), nor maternal (*n* = 9 good condition, *n* = 3 poor condition) or calf body condition categories (*n* = 5 good condition, *n* = 7 poor condition), although this may relate to the study’s limited sample size. By linking endocrine profiles with body condition, this study provides novel physiological context for understanding maternal investment strategies in southern right whales. The findings highlight how intra-seasonal energetic demands are hormonally mediated during lactation and demonstrate the feasibility of integrating photogrammetry with endocrine monitoring in free-swimming baleen whales. Establishing these physiological baselines is critical for detecting early indicators of reproductive stress and for informing conservation strategies in recovering populations.

## Introduction

Reproduction in long-lived, iteroparous and K-selected mammals requires careful management of energetic resources due to their substantial investment in only a small number of offspring (LaSharr *et al.,* 2025). In these species, maternal investment strategies reflect trade-offs between maintaining their own condition for survival and providing for their dependent young (Stearns, 1998). These investment strategies, however, vary depending on how energy is acquired and stored. In capital-breeding species that rely entirely on previously accumulated reserves rather than continuous foraging to fuel the energetically demanding stages of reproduction (Jönsson and Jonsson, 1997), the accumulation and management of their energy stores is central to their reproductive success.

Southern right whales (*Eubalaena australis*; SRWs) are an example of a capital breeding baleen whale species. During intensive summer foraging seasons, they must accumulate a thick blubber layer, rich in lipids (Lockyer, 1981), to sustain the energetic demands of basic metabolic function, migration to calving grounds and different phases of reproduction. In reproductively active females, this energetic buffer is critical especially for late gestation and lactation, during which females can lose up to 25% of their body volume (Christiansen *et al.,* 2018). Considering the high demands of reproduction, it is unsurprising that SRW reproductive success is tightly coupled to foraging success prior to the reproductive period, as energy reserves acquired during the feeding season fuel gestation and lactation. Indeed, studies show how climate-induced fluctuations in their primary prey source, Antarctic krill (*Euphausia superba*) influence reproductive output (Leaper *et al.,* 2006; Seyboth *et al.,* 2016).

Understanding the physiological mechanisms that underpin maternal energy balance is therefore crucial, particularly during the energetically demanding lactation phase, as it allows for assessing their capacity to successfully provision and wean their calves. Given that calves in an optimal condition have a higher likelihood of survival and demonstrate greater resilience to environmental variability (Christiansen *et al.,* 2018; Pettersen *et al.,* 2022; Adamczak *et al.,* 2023), it ultimately allows for the evaluation of population health and viability. Nonetheless, to date, such physiological mechanisms remain poorly understood, due to the logistical challenges associated with studying free-swimming large whales (McHuron *et al.,* 2022). Technological advances and multidisciplinary approaches, however, provide the opportunity to bridge these gaps and deepen our understanding of baleen whale physiology (Costa and Favilla, 2023; Bruno, 2024) as illustrated by studies combining UAV photogrammetry with blubber lipid concentration in biopsies (Christiansen *et al.,* 2020) or with faecal hormone analyses (Pirotta *et al.,* 2023).

Body condition, defined as any physiological index that can be used to determine an individual’s energy reserves (Millar and Hickling, 1990; Castrillon and Bengtson Nash, 2020), and which can be measured through photogrammetric methods (Christiansen *et al.,* 2013, 2023; Napoli *et al.,* 2024; van Aswegen *et al.,* 2025), provides a useful baseline for understanding energetics by providing a quantifiable metric for energy availability. However, in the context of lactation, it does not provide information on the internal regulatory processes that govern energy mobilisation and expenditure. Therefore, additional biochemical assessments like hormone quantification can aid in improving our understanding of the physiology of lactation in SRWs.

Endocrine analysis has become an increasingly valuable tool in conservation physiology, gaining popularity as a method to identify endocrine correlates of reproductive function and responses to stressors. Hormone concentrations can be measured in a variety of matrices (Hunt *et al.,* 2014, 2017, 2019) with blubber being the most logistically feasible matrix for free-swimming baleen whales, which can be obtained from minimally invasive biopsy sampling (de Mello and de Oliveira, 2016; Melica *et al.,* 2021). Glucocorticoids (GCs), including cortisol and corticosterone, and thyroid hormones, including triiodothyronine (T₃) and thyroxine, are two classes of hormones that can effectively be quantified to enhance our understanding of animal energetics; while GCs are associated with stress, particularly nutritional stress in this context, they play a crucial mechanistic role in energy mobilisation, especially of lipids (Fowler *et al.,* 2016; Fernández Ajó *et al.,* 2020; Melica *et al.,* 2022). Complementary, thyroid hormones, which are indicative of metabolic rate (Fernández Ajó *et al.,* 2020), can help to disentangle the causes of upregulated metabolic activity. Both cortisol and corticosterone have been shown to produce biologically plausible patterns across time for baleen whales (Hunt *et al.,* 2018). However, T_3_ is considered the more biologically active thyroid hormone (Eales, 1988; Fernández Ajó *et al.,* 2020). Therefore, in this study, predominantly cortisol and T_3_ were selected to be quantified. Through the combination of photogrammetry and multi-hormone endocrine analyses, this study aims to provide a baseline assessment of metabolic function in lactating SRWs over the calving season.

The specific objectives of the research are to (a) compare GC and T_3_ concentrations between lactating females and non-lactating adults (so called ‘unaccompanied adults’), (b) assess intra-seasonal changes in GC and T_3_ concentrations in lactating females, and (c) evaluate GC and T_3_ concentrations of lactating females relative to their own body condition and (d) the body condition and (e) length of their calves. Together, these analyses provide a more holistic understanding of maternal investment strategies in SRWs. By integrating photogrammetry with endocrine markers, this study combines external and internal metrics, demonstrating how a multi-disciplinary approach can enhance monitoring of maternal investment and reproductive performance in wild populations of baleen whales.

## Materials and Methods

### Ethics statement

The following research was completed under University of Pretoria ethics permit numbers NAS305/2021 and NAS067/2023.

### Fieldwork

Boat-based fieldwork was conducted between July and September of 2022, 2023 and 2024 in Walker Bay, South Africa (34.50°S, 19.33°E) using a semi-rigid 6 m vessel. Fieldwork was carried out in good weather conditions (Beaufort sea state <3) by a team of 4–5 experienced researchers, with the sole purpose to collect UAV videos and biopsy samples of SRWs.

#### Video data collection

Overhead video recordings were obtained using a DJI Mavic 2 Zoom unmanned aerial vehicle (UAV) operated by a licensed pilot at altitudes between approximately 5 and 35 meters under calm sea conditions (Beaufort sea state <3). Specifications of the UAV include an unfolded span of 322 mm and mass of 905 g, featuring a 1/2.3″ CMOS sensor with 12-megapixel resolution (4000 × 3000 pixels) and a zoom lens ranging from 24 to 48 mm. For precise altitude tracking, the UAV was equipped with a custom laser range finder (SF11/C; ~ 109 g; powered by a 500 mAhr LiPo battery; resolution: 1 cm; accuracy: ±0.1 m). Recording duration varied depending on how long it took for the whale to settle in a horizontal position at the surface, with its dorsal side exposed and body axis straight (Christiansen *et al.,* 2016). A real-time video feed transmitted to an iPhone 7 via the drone’s camera allowed the operator to maintain visual contact and adjust positioning as needed.

#### Biopsy sample collection

Blubber biopsy samples were collected from both calf and mother using a Barnett Panzer V crossbow (150 lb draw weight), equipped with biopsy darts featuring hollow stainless-steel tips designed to extract skin and blubber while limiting penetration depth to 20 mm via an internal stopper, as outlined in Krützen *et al.* (2002). For this, animals were approached laterally at a controlled speed of 1–4 knots. Up to three biopsy dart deployment attempts (i.e. dart firings) were permitted per individual. Once retrieved, biopsy tips containing tissue were stored in tubes placed in a cooler with reusable ice packs for the remainder of the field day. Upon return to land, samples were removed from the tips, separated into skin and blubber components, and stored at −20°C for subsequent laboratory analysis.

### Sample selection criteria

Using the aerial footage, still frames of each individual, identified through their unique callosity pattern (Payne *et al.,* 1983), were taken where the animal lay unobstructed and dorsal side up. These images were quality graded based on camera focus, body orientation (roll, pitch and arch), and visibility of key anatomical features (rostrum, fluke notch, body outline) according to the protocol established by Christiansen *et al.* (2018). Only complete sampling events (which included high-quality UAV imagery of both the mother and calf, and a maternal blubber sample for endocrine analysis) were prioritised for analysis. In the event of an individual being sampled multiple times in a calving season, the complete sampling event with the highest quality images was used to avoid pseudoreplication (as the number of repeated measures was insufficient to account for statistically.) A body length threshold (>12.4 m; Best and Rüther, 1992) was used to ensure that juveniles were not included as unaccompanied adults.

### Body condition assessment

Morphometric measurements (distance from the tip of the rostrum to the notch of the fluke, location of the eyes and end of the tail stock as well as body widths taken at 5% intervals perpendicular to the body axis) were extracted from selected still images, first in pixels and then converted to meters using a custom R script (R version 4.3.2; R Core Team, 2022), following the methodology outlined by Christiansen *et al.* (2016). Images were taken at a resolution of 4608 × 3456 pixels, the camera sensor dimensions were 7.17 × 4.55 mm, and the focal length of the camera lens was 4.49 mm. Body volume was calculated using these distance measurements by modelling the cross-sectional shape at each 5% interval as a series of infinite ellipses formed between adjacent segments using published paired width and height values following Christiansen *et al.* (2019). Finally, the volume measurements were used to calculate body condition as described in Christiansen *et al.* (2018), using the south–western population of Australia as a reference for healthy individuals (Christiansen *et al.,* 2018, 2020). A positive body condition score indicates an individual is in better than average condition relative to its population, while a negative score suggests poorer condition (Christiansen *et al.,* 2018). Note that throughout this study, the terms ‘good’ and ‘poor’ are used to refer to positive and negative body condition scores, respectively, relative to the reference population and are not intended as absolute indicators of health.

### Endocrine analysis

All laboratory procedures were conducted in the Endocrine Research Laboratory, University of Pretoria, South Africa.

#### Blubber hormone extraction

Hormones were extracted from blubber samples following a modified version of a previously established protocol (Kellar *et al.,* 2006). The modifications, in brief, included homogenising samples in 1.5 ml of 100% ethanol for 4 cycles of 50 seconds, with each cycle 30 seconds apart. The homogenate was reextracted with an additional 1.5 ml of 100% ethanol, vortexed, and then centrifuged for 15 minutes at 1500 g. The ethanol extract was transferred to a glass tube and blubber residue was resuspended in 3 ml of 4:1 ethanol-acetone instead of diethyl ether for an additional cycle of extraction. Both ethanol and ethanol-acetone extracts were mixed together and dried until no solvent was seen. The obtained precipitate was further extracted by biphasic acetonitrile-hexane separation using a volume of 1.5 ml of each solvent followed by vortex and centrifugation at 1500 g. The mixture was dried and stored at −20° until hormone quantification.

#### T3 quantification

Blubber T_3_ was quantified using a commercially available enzyme immunoassay (EIA) (K056-H5, Arbor Assay, USA). The assay has previously been used to successfully quantify T_3_ in SWR baleen (Fendandez Ajo et al. 2020). Dried hormone precipitate was reconstituted in 0.5 ml of 100% ethanol, followed by vortexing. The intra-assay precision ranged from 5.5 to 6.7%. The sensitivity of the EIA was determined as 37.4 pg/ml. Note that the precision and sensitivity values are according to the user manual provided by the manufacturer. All samples were quantified using a 1:20 dilution; therefore, parallelism was not tested.

#### GC quantification

Glucocorticoid concentrations in the blubber extracts were determined using a cortisol EIA (Palme and Möstl, 1997). Note that this EIA makes use of polyclonal antibodies, which quantify immunoreactive glucocorticoids. While cortisol is the predominant glucocorticoid detected, the antibodies show limited cross-reactivity with corticosterone (6.2%), such that measured values primarily reflect cortisol concentrations with minor contribution from other GCs. For this, 0.05 ml of the steroid extract was dried down in an oven at 50°C for 60 minutes and reconstituted in 0.25 ml of assay buffer prior to analysis. Intra-assay coefficients of variation (CV), determined by quantifying diluted standard-steroid high and low-quality controls (*n* = 20 each), were 4.81 and 5.62%. The sensitivity of the cortisol EIA was 0.08 ng/g of blubber mass. Antibody cross-reactivities are provided in Palme and Möstl (1997). All samples were quantified using a 1:5 dilution.

#### Biological assay validation

To evaluate the biological plausibility of the GCs and T_3_ assays used, hormone concentrations from a confirmed pregnant female were determined, as higher GC and T₃ concentrations can be expected during late pregnancy compared to the lactation period. This female was observed early in the calving season as an unaccompanied adult and later resighted (confirmed through photo identification; Payne *et al.,* 1983) with a calf. This female yielded markedly elevated concentrations of both GCs and T_3_ (18.43 and 200.10 ng/g blubber, respectively), that were approximately ten- and fivefold higher than the mean of all other measured samples. Although this observation does not constitute a formal assay validation, it provides qualitative biological context, which is particularly valuable in baleen whale endocrine studies where traditional assay validation is often not feasible.

### Data analysis

The dataset was assessed for normality using Shapiro–Wilk’s test. Correlation between hormones was assessed using Spearman’s Rank test. A Wilcoxon rank-sum and two-sample *t*-test were used to compare differences in T_3_ and GC concentrations of (i) lactating females and unaccompanied adults (objective a), (ii) females of different body condition categories (good = positive body condition score, poor = negative body condition score; objective c), and (iii) females with calves of different body condition categories (good = positive body condition score, poor = negative body condition score; objective d). To assess intra-seasonal changes in hormone concentrations of lactating females, a Kruskal–Wallis with post-hoc Dunn’s test (Holmes correction) and ANOVA test were used to compare GC and T_3_ levels across different months, respectively (objective b). Linear models and generalised additive models (GAMs) were used to assess potential linear and non-linear relationships between calf length and maternal hormone concentrations (objective e). Separate models were fitted for GCs and T_3_ as response variables, with calf length as the predictor. The full dataset excluding the pregnant female used as a biological validation (*n* = 16 lactating females, *n* = 7 unaccompanied adults) was used for most analyses to maximise statistical power. However, due to insufficient quality of some images for morphometric measurements, data from four lactating females were excluded from analyses involving body condition and calf length (*n* = 12 lactating females) but retained where appropriate in broader comparisons where only endocrine data was assessed. Although classed as an unaccompanied adult, the pregnant female was excluded from analyses so as not to skew results, given the measured hormone concentrations in this sample were considered outliers.

## Results

### Sampling effort

Across the three field seasons combined, a total of 32 research days and 101 UAV flights were completed, spanning 69 Julian days between the first and last survey dates (17 June–28 September). During the flights, 216 non-unique individual SRWs were video recorded, from which still frames were taken for further analysis. Of these, 131 individuals were biopsied. Based on the sample selection criteria, which required a complete sampling set including photogrammetry data for both mother and calf and a corresponding blubber biopsy, biopsies from 16 lactating females and 8 unaccompanied adults were sent for endocrine analysis representing samples for *n* = 9 in 2022, *n* = 4 in 2023, and *n* = 11 in 2024.

### Demographic differences in hormone concentrations

Both GC and T_3_ concentrations were detected in the blubber samples of lactating female and unaccompanied adult SRWs. Summary statistics of hormone concentrations are presented in Table 1. No significant differences in GC (W = 83, *P* = 0.08, *n* = 24) nor T_3_ (*t* = −0.61, df = 14.35, *P* = 0.55, *n* = 24) concentrations were detected between lactating females and unaccompanied adults. Visually, however, both mean and median GC concentrations were slightly higher in lactating females, while mean and median T_3_ concentrations were slightly higher in unaccompanied adults.

**Table 1:** Descriptive statistics including mean, standard deviation (SD) and 95% confidence intervals (95%CI) for glucocorticoids (GC) and triiodothyronine (T_3_) measured in lactating female and unaccompanied adult southern right whales sampled in Walker Bay, South Africa over 2022, 2023 and 2024

	GC (ng/g)			T_3_ (ng/g)		
	Mean	SD	95% CI	Mean	SD	95% CI
Overall (*n* = 23)	1.75	1.08	[1.28, 2.21]	40.63	19.27	[32.30, 48.97]
Lactating females (*n* = 16)	1.90	1.04	[1.35, 2.46]	39.12	20.68	[28.10, 50.14]
Unaccompanied adults (*n* = 7)	1.38	1.17	[0.30, 2.57]	44.10	16.51	[28.83, 59.38]

An overall moderate positive correlation was found between GC and T_3_ concentrations (Spearman’s ρ = 0.44, *P* = 0.038, *n* = 23). A slightly stronger correlation was detected when the dataset was restricted to lactating females (Spearman’s ρ = 0.65, *P* = 0.008, *n* = 16), while a much weaker and non-statistically significant relationship was observed in unaccompanied adults (Spearman’s ρ = 0.18, *P* = 0.71, *n* = 7).

### Intra-seasonal variation in hormone concentrations among lactating females

**Figure 1:**
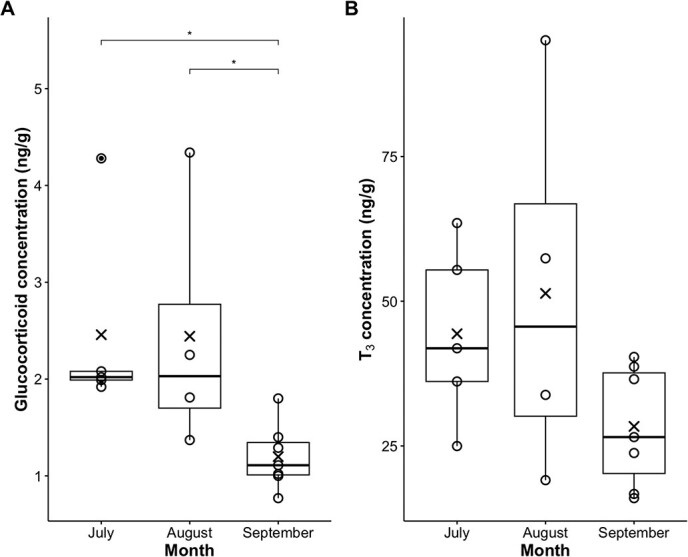
Boxplots of glucocorticoid (A) and triiodothyronine (B) concentrations measured in lactating female southern right whales (*n* = 12) sampled in Walker Bay, Hermanus, over the calving season (July—September). Boxes represent the 25th and 75th percentiles, the horizontal line indicates the median, X’s denote the mean, whiskers denote the range, and solid dots represent outliers. Measured datapoints are shown as open circles. Significant differences among months are indicated by an asterisk (*: *P* < 0.05).

In lactating females, a significant difference was observed in GCs (χ^2^ = 9.84, df = 2, *P* = 0.007, *n* = 16; Fig. 1A) over the sampling period (July: *n* = 5, Aug: *n* = 4, Sept: *n* = 7), but not in T₃ (χ^2^ = 3.09, df = 2, *P* = 0.21, *n* = 16; Fig. 1B). Post hoc Dunn tests with Holm correction revealed that GC concentrations were significantly lower in September compared to both August (*Z* = 2.33, *P* = 0.039) and July (*Z* = 2.84, *P* = 0.014), while no difference was found between July and August (*Z* = −0.30, *P* = 0.77). Although statistically not significant, T₃ concentrations also seemed to decline over the calving season.

### Hormone concentrations in relation to maternal and calf body condition

**Figure 2:**
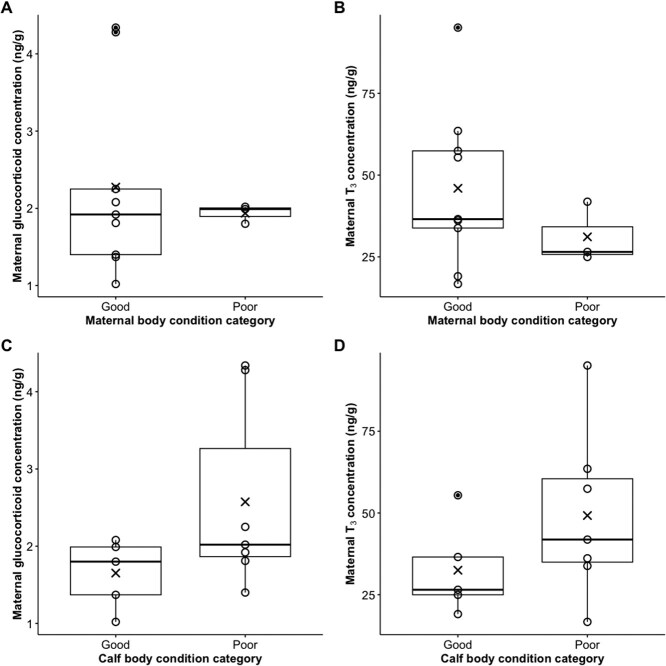
Boxplots of glucocorticoid (A and C) and triiodothyronine (T_3_) (B and D) concentrations measured in lactating female southern right whales (*n* = 12) sampled in Walker Bay, South Africa, during the calving season (July—September). Hormone concentrations are shown in relation to maternal body condition (A and B) and calf body condition (C and D). Boxes represent the 25th and 75th percentiles, the horizontal line indicates the median, X’s denote the mean, whiskers denote the range, and solid dots represent outliers. Measured datapoints are shown as open circles.

Summary statistics of body condition measurements are detailed in Table 2. No significant difference was found in GC (*W* = 14, *P* = 1.00, *n* = 12; Fig. 2A) nor T₃ (*t* = 1.51, df = 9.40, *P* = 0.16, *n* = 12; Fig. 2B) concentrations among lactating females in either good or poor body condition categories. Nonetheless, a greater range in both GCs and T₃ concentrations was measured for lactating females in good body condition, and these females also had a 48% higher mean T₃ concentration (Fig. 2B).

**Table 2:** Descriptive statistics including mean, standard deviation and range for body condition scores measured in southern right whale lactating females and calves sampled in Walker Bay, South Africa over 2022, 2023 and 2024. Good condition includes body condition scores >0 while poor condition includes body condition scores <0

	Mean	Standard deviation	Range
Lactating females			
Overall (*n* = 12)	0.05	0.09	−0.08 to 0.26
Good condition (*n* = 9)	0.08	0.08	
Poor condition (*n* = 3)	−0.02	0.03	
Calves			
Overall (*n* = 12)	−0.01	0.10	−0.20 to 0.17
Good condition (*n* = 5)	0.08	0.03	
Poor condition (*n* = 7)	−0.08	0.06	

Similarly, no difference was found in GC and T_3_ concentrations of females accompanied by calves in either good or poor body condition (GCs: *W* = 8, *P* = 0.15, *n* = 12; T₃: *t* = −1.45, df = 9.64, *P* = 0.18, *n* = 12; Fig. 2C and D). Nonetheless, a greater range in maternal GC and T₃ concentrations was observed in females with calves in poor body condition, and these females had a 55.8 and 51.4% higher mean in GC and T_3_, respectively (Fig. 2C and D).

### Maternal hormone concentrations in relation to calf length

Linear models showed no association between calf length and GC concentrations (adjusted *R*^2^ = −0.054, *F*_1,10_ = 0.44, *P* = 0.52) or T_3_ concentrations (adjusted *R*^2^ = −0.01, *F*_1,10_ = 0.89, *P* = 0.37). Generalised additive models likewise indicated no evidence of non-linear relationships (GC: adjusted *R*^2^ = −0.054, deviance explained = 4.21%, edf = 1; T_3_: adjusted *R*^2^ = −0.01, deviance explained = 8.18%, edf = 1).

## Discussion

This study showed a clear intra-seasonal trend in metabolic hormonal regulation in adult females during the lactation period, with both GCs and T₃ declining near the end of the calving season, albeit the latter not significantly so. This suggests a reduction in maternal metabolic activity as the calving season progresses and calf growth rates slow, highlighting that endocrine profiles may track intra-seasonal changes in energetic regulation during lactation. Interestingly, hormone concentrations did not appear to be linked to demographic group, nor to maternal or calf body condition, or calf length. However, the limited sample size may hamper further interpretation of these results and continuation of this research with increased sample size may reveal different patterns in future. Regardless, the observed patterns suggest that endocrine regulation at least in lactating females may be driven by intra-seasonal energetic constraints rather than individual variability reflecting a consistent maternal investment strategy among lactating females.

Hormone concentrations were expected to differ between demographic groups in line with their differing energetic requirements, with higher concentrations for both hormones expected to be measured in lactating females (Crespi *et al.,* 2013; Fortune *et al.,* 2013; Zwahlen *et al.,* 2024). There was, however, no significant difference detected in either GC or T₃ concentrations between unaccompanied adults and lactating females in this study. For GCs, this is similar to observations in blue whales (*Balaenoptera musculus)* where sex and reproductive state did not influence patterns in in blubber GCs (Atkinson *et al.,* 2020; Melica *et al.,* 2022). However, it contrasts the patterns observed in grey whales (*Eschrichtius robustus*) where lactating females displayed higher GC levels than any other non-calving reproductive group (Melica *et al.,* 2022; Pirotta *et al.,* 2023). This highlights the complexity and species-specific role that GCs play in mediating the energetics of different mysticetes. Future work incorporating sex differentiation of unaccompanied adults would help further resolve potential sources of variation in GC and T₃ concentrations among demographic groups.

The positive correlation between GCs and T₃ detected in both unaccompanied adults and lactating females suggests coordinated endocrine regulation of lipid mobilisation and metabolism during time spent on coastal grounds. However, the substantially stronger correlation detected among lactating females suggests that this relationship is likely more functionally important for supporting the elevated energetic demands of lactation in females than for the basic metabolic functions of unaccompanied adults. While there is a general assumption that thyroid hormones and GCs are antagonistic (Ayres *et al.,* 2012; Gobush *et al.,* 2014), T₃ and corticosterone were shown to be positively correlated in the baleen of bowhead whales (*Balaena mysticetus;*  Hudson *et al.,* 2025). This observation led to the conclusion that, for mysticetes, certain stressors indicated by upregulated GCs may require an increased metabolic rate, as reflected in the coinciding increase in T₃ (Hudson *et al.,* 2025). If lactation is perceived as a stressor, this may explain the positive correlation in hormones observed in this study.

Although lactation places substantial energetic strain on females (Christiansen *et al.,* 2018), the observed patterns in GCs and T₃ likely reflect normal physiological regulation rather than pathological stress. GCs are known to have a catabolic function, playing a crucial role in lipid mobilisation and gluconeogenesis in baleen whales (Melica *et al.,* 2022), providing the necessary energy for milk synthesis. This has been demonstrated in other marine mammals, such as northern elephant seals (*Mirounga angustirostris*) where maternal cortisol levels were shown to impact the lipid content of their milk (Fowler *et al.,* 2016). Thyroid hormones, while poorly understood in baleen whales, are known to play a role in the regulation of metabolic activity, growth, development, thermoregulation, and migratory physiology (Hudson *et al.,* 2025). In the context of lactation, although, thyroid hormones are generally suppressed during fasting as a way to slow metabolic rate and decrease energy expenditure, thyroid-promoted lipolysis can further help meet energetic demands during prolonged periods of nutritional stress (Lysiak *et al.,* 2018). Together, these endocrine mechanisms facilitate the conversion of stored maternal energy into lipid-rich milk to meet the nutritional needs of their calves while simultaneously maintaining their own metabolic requirements, and that these hormonally mediated shifts help maintain energetic balance during periods of fasting, typical for capital breeders.

As K-selected, iteroparous species, baleen whales are likely to prioritise their own survival over that of their calf (MacArthur and Wilson, 1967; Hamel *et al.,* 2010). Individuals in poor body condition are therefore expected to downregulate metabolic activity to conserve energy. Lower blubber GCs have previously been detected in poorer condition individuals in SRWs near the end of the calving season (Thavar *et al.,* 2021) and in humpback whales (*Megaptera novaeangliae*) during late stages of migration (Dalle Luche *et al.,* 2021). In this study, median GC and T₃ values were similar for lactating females irrespective of body condition. However, the broader range observed in females in good condition suggests greater physiological flexibility in individuals with more energy reserves. Conversely, females in poorer condition may exhibit more constrained hormonal regulation, consistent with a limited energetic capacity. When relating maternal hormone concentrations to calf body condition, females with poorer condition calves showed slightly elevated hormone levels, possibly reflecting upregulated metabolic output when provisioning demands are high. Yet, the co-occurrence of poor calf condition suggests these compensatory efforts may be insufficient or may represent a time lag between the upregulation of maternal metabolism and the effects being reflected in calf growth.

Abnormal energetic constraints, resulting, for example, from severe nutritional deficiency, reflected in elevated GCs or suppressed T₃ concentrations were not apparent in the measured subset of the study population and are in line with other long-term observations. As this study took place at coastal calving grounds, the sampled individuals were presumed to be those physiologically capable of sustaining the energetic demands of migration, and for reproductively active females, late gestation and lactation. For unaccompanied adults, the marked decline in their coastal presence at South African calving grounds since 2009 (Brandão *et al.,* 2023; Vermeulen *et al.,* 2025), suggests that only animals with sufficient energetic reserves continue to undertake coastal migration. Similarly, females will only successfully birth a calf and undergo lactation if they are in adequate body condition (Christiansen *et al.,* 2020). If not, they are likely to either delay conception or will experience late-term foetal abortion or early calf mortality (Knowlton *et al.,* 1994) as is reflected by increased prevalences of 4- and 5-year calving intervals (Brandão *et al.,* 2023; Vermeulen *et al.,* 2025). Consequently, individuals in the poorest physiological state are unlikely to appear in coastal nursery areas and would therefore not get sampled.

Body condition is subject to substantial change (Christiansen *et al.,* 2016) and should not be used in isolation when investigating the physiology and endocrine regulation of lactation. Both maternal and calf body condition follow intra-seasonal trends, with female body condition in the investigated population being predominantly positive at the start of the calving season and negative near the end. Calves display the opposite trend. The clear temporal decline, particularly from August to September, in both GC and T₃ (although the latter not statistically significant) concentration among lactating females reflects these changes in energetic demands over the calving season and aligns with expectations for a capital-breeding species as well as with known curvilinear growth patterns of SRW calves (Christiansen *et al.,* 2018, 2022). Results of this study suggest that females elevate metabolic activity early in lactation when calf growth is rapid and most demanding energetically, which coincides with times of abundant maternal energy reserves. As calves mature and maternal energy becomes more constrained, maternal metabolic activity is then reduced. This once more is in line with reproductive strategies of iteroparous and K-selected species where a balance is struck between provisioning for a calf up to the point where their energetic demands are met, after which investment is reduced to avoid compromising maternal survival, as is seen during foetal development of minke whales (*Balaenoptera acutorostrata*; Christiansen *et al.,* 2013).

By linking endocrine profiles with body condition, this study documents intra-seasonal endocrine variation during lactation, offering baseline physiological context for understanding maternal investment in SRWs. Future work with larger sample size will be need to clarify how individual reserves are hormonally regulated. This study provides a foundational baseline representing the first integration of photogrammetry with blubber GC and T₃ measurements**,** and the first assessment of blubber T₃ in free-swimming SRWs, a hormone which, for this species, has previously only been quantified retrospectively in baleen (Lysiak *et al.,* 2018; Fernández Ajó *et al.,* 2020). It further builds on our knowledge of GCs in the South African population, which have only been preliminarily assessed (Thavar *et al.,* 2021). Finally, the study demonstrates the feasibility and utility of multidisciplinary research for furthering our understanding of reproductive physiology of baleen whales. While continued long-term sample collection is recommended, establishing these physiological baselines is critical for understanding present-day reproductive strategies and detecting early warning indicators of future reproductive stress.

## Data Availability

The data underlying this article will be shared on reasonable request to the corresponding author.
